# Malignant Hepatic Infiltration Versus Drug-Induced Liver Injury in B-Cell Acute Lymphoblastic Leukemia

**DOI:** 10.14309/crj.0000000000002234

**Published:** 2026-07-16

**Authors:** Jeffrey Liu, Arpine Petrosyan, Jennifer Yoon, Marina Roytman

**Affiliations:** 1 Department of Internal Medicine, University of California San Francisco School of Medicine, Fresno, CA; 2 Division of Gastroenterology and Hepatology, University of California San Francisco School of Medicine, Fresno, CA

**Keywords:** DILI, lymphocytes, liver, lymphoblastic leukemia

## Abstract

B-cell acute lymphoblastic leukemia (B-ALL) is the most common subtype of lymphoid malignancy. Elevated liver enzymes in B-ALL may reflect both hepatic infiltration and chemotherapy-related drug-induced liver injury. This creates a diagnostic challenge that can be delineated with a liver biopsy, preventing delays in management and care. We present 2 patients with B-ALL who developed elevated liver enzymes during chemotherapy and underwent liver biopsy demonstrating hepatic infiltration. These biopsy-proven cases highlight the critical role of liver biopsy in distinguishing hepatic infiltration from drug-induced liver injury, guiding management decisions and allowing for continued chemotherapy without unnecessary delay.

## INTRODUCTION

In acute lymphoblastic leukemia (ALL), malignant proliferation of immature lymphoblasts arises from hematopoietic progenitors, with more than 70% of adult cases being B-cell (B-ALL) in origin.^1–3^ Both hepatic lymphoblastic infiltration and chemotherapy-related drug-induced liver injury (DILI) may present with elevated transaminases, creating a diagnostic challenge.^4,5^ We present 2 cases of elevated transaminases during chemotherapy in which liver biopsy distinguished hepatic lymphoblastic infiltration from DILI.

## CASE REPORT

### Case 1 presentation

A 19-year-old Hispanic woman presented to the hospital with headaches and witnessed seizure. She reported 2 months of nausea, weight loss, anorexia, and abdominal pain. She denied any alcohol, supplement, or illicit drug use. She was recently diagnosed with CD20^+^ B-ALL on bone marrow biopsy and started on prednisone while awaiting the start of chemotherapy. After initiation of pegaspargase and rituximab, treatment was paused due to elevated transaminases, with alanine aminotransferase and aspartate aminotransferase peaking at 320 and 161 IU/L, respectively (Table 1).

**Table 1.** Timetable of transaminases with initiation of rituximab in case 1

Case 1	Value (IU/L)
Pretreatment laboratory test results	
Alanine aminotransferase	12
Aspartate aminotransferase	22
Alkaline phosphatase	79
Rituximab day 1	
Alanine aminotransferase	210
Aspartate aminotransferase	83
Alkaline phosphatase	120
Status post Rit day 3	
Alanine aminotransferase	320
Aspartate aminotransferase	161
Alkaline phosphatase	127
Status post Rit day 5	
Alanine aminotransferase	307
Aspartate aminotransferase	88
Alkaline phosphatase	136

Rit, Rituximab.

**Figure 1.**
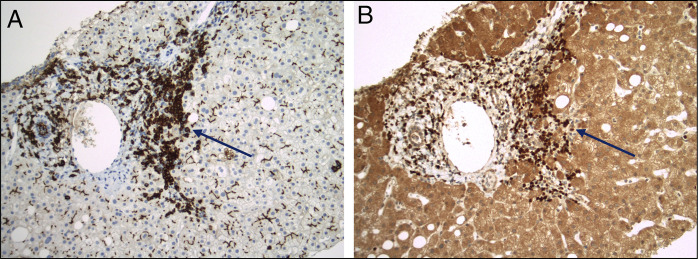
Immature lymphoblasts surrounding a portal tract without duct destructive lesions nor hepatocyte apoptosis. (A) CD10^+^ staining (B) TDT^+^ staining. 200× magnification. Blue arrows highlighting lymphoblastic infiltration within hepatic parenchyma.

Her R-factor score was 6.8 suggesting hepatocellular injury. Her chemotherapy was intermittently halted due to concerns for DILI. Hepatology was consulted and imaging showed hepatosplenomegaly. A liver biopsy found mild portal tract expansion by mononuclear cells without duct-destructive lesions, cholestasis, or hepatocyte apoptosis. Immunohistochemical staining showed TdT/CD10/CD34^+^ mononuclear cells consistent with lymphoblastic infiltration (Figure 1). Given biopsy-proven hepatic B-ALL infiltration and DILI was less likely, her chemotherapy was resumed, with her transaminases returning to baseline 11 days from initiating rituximab.

### Case 2 presentation

A 24-year-old Hispanic man was transferred from outside hospital for induction chemotherapy for his newly diagnosed B-ALL. He had presented with a dry cough but denied fevers or chills. He also denied any alcohol, supplements, or illicit drug use. His laboratory workup showed leukocytosis of 36.9 ×10^3^/µL and thrombocytopenia of 91 ×10^3^/µL. His bone marrow biopsy from the outside hospital found B-ALL with partial CD20^+^ cells. He was given doses of cyclophosphamide, vincristine, and doxorubicin; however, his chemotherapy was paused due to a significant rise in his transaminases, with alanine aminotransferase and aspartate aminotransferase peaking at 487 and 213 IU/L, respectively (Table 2).

**Table 2.** Timetable of transaminases with initiation of intrathecal cytarabine, cyclophosphamide, vincristine, & doxorubicin in case 2

Case 2	Value (IU/L)
Intrathecal cytarabine	
Alanine aminotransferase	487
Aspartate aminotransferase	213
Alkaline phosphatase	97
Status post IT Cyt day 8/Cyc day 1	
Alanine aminotransferase	131
Aspartate aminotransferase	45
Alkaline phosphatase	73
Cyc day 4/Vin & Dox day 1	
Alanine aminotransferase	125
Aspartate aminotransferase	26
Alkaline phosphatase	70
Status post Cyc day 6/s/p Vin & Dox day 3	
Alanine aminotransferase	262
Aspartate aminotransferase	71
Alkaline phosphatase	59

Cyc, cyclophosphamide; Cyt, cytarabine; Dox, doxorubicin; IT, intrathecal; Vin, vincristine.

**Figure 2.**
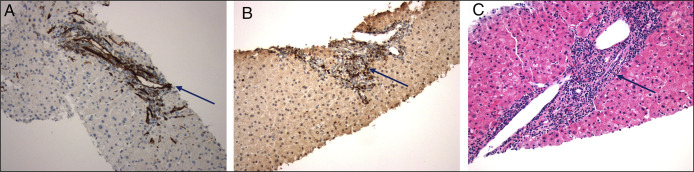
Immature lymphoblasts and periportal infiltration without evidence of hepatocyte necrosis nor cholestasis. (A) CD10^+^ staining (B) TDT^+^ staining (C) Hematoxylin and eosin staining. 200× magnification. Blue arrows highlighting lymphoblastic infiltration within hepatic parenchyma.

Hepatology was consulted, and liver biopsy showed portal areas with scattered CD34^+^/Ki-67^+^ nonendothelial cells in portal areas, a lack of CD20^+^ cell expansion, and no evidence of cholestasis or apoptosis. Although liver biopsy found lymphoid infiltration, DILI was still possible based on his Roussel Uclaf Causality Assessment Method (RUCAM) score of 3. As he continued chemotherapy his transaminases returned to normal limits 12 days after initiating vincristine (Figure 2).

## DISCUSSION

In these 2 cases, both patients were diagnosed with B-ALL by bone marrow biopsy and flow cytometry demonstrating B-cell lineage markers (CD10, CD20, TdT, and CD34). Although B-ALL primarily involves bone marrow, with extramedullary involvement rarely includes the liver and spleen.^5–7^ During chemotherapy, both patients developed elevated transaminases initially concerning for chemotherapy-related DILI, resulting in interruption of treatment while further evaluation was pursued. Viral hepatitis, alcohol-related liver injury, and other common etiologies were excluded; however, DILI and hepatic lymphoblastic infiltration remained in the differential diagnosis. Liver biopsy in both cases demonstrated hepatic infiltration, highlighting a diagnostic challenge in the management of B-ALL. Notably, these cases represent the key diagnostic value of liver biopsy in identifying lymphoblastic infiltration during active chemotherapy, directly informed management decisions and supporting continuation of treatment.

Chemotherapeutic agents are well known causes for DILI, complicating evaluation of hepatocellular injury. To assess chemotherapy-related injury, a RUCAM analysis was performed (Table 3). Rituximab is associated with moderate transaminase elevations, although severe hepatotoxicity (5 times the upper limit of normal) is uncommon, occurring in 0.5%–1.5% of patients.^8^ Hepatic injury is most related to hepatitis B virus reactivation. In our first case, biopsy-proven lymphoblastic infiltration and negative hepatitis B virus serologies made rituximab-induced injury unlikely. Cyclophosphamide may cause mild, transient transaminase elevations in up to 43% of patients, with rare cases of acute liver injury reported, typically in older patients with more comorbid conditions.^9^ Injury usually occurs within 10–20 days of therapy initiation and is characterized histologically by sinusoidal cell necrosis and hepatic vein obliteration. Doxorubicin may also cause transient, asymptomatic transaminase elevations, often without progression despite continued therapy.^10^ Vincristine-associated hepatotoxicity is rare and difficult to isolate because it is typically administered in combination regimens.^11^ In our second case, biopsy-confirmed hepatic infiltration strongly supported lymphoblastic hepatic involvement, although a RUCAM score of 3 suggested possible contribution from vinca alkaloid-related DILI.

**Table 3.** RUCAM analysis scores for chemotherapeutic agents in Case 1 & Case 2

Causality assessment	Hepatocellular	Points assigned
**Case 1**		
Medication(s) and dose	Rituximab - 600 mg	—
Timing	4 days before elevation	*1*
Timing	Medication continued	0
Other risks	None identified	0
Literature data	Reactivation of HBV	*1*
Exclusion of other Group causes	Group 1 excluded	*1*
	Group 2 excluded (biopsy proven)	0
Reexposure	No further elevations	*−2*
Total	—	**1**
**Case 2**		
Medication(s) and dose	Cyclophosphamide - 540 mg	—
	Vincristine - 2 mg	—
	Doxorubicin - 90 mg	—
Timing	Cyclophosphamide - 5 d	0
	Vincristine/Doxorubicin - 2 d	*1*
Timing	Elevation of enzymes before administration	0
Other risks	None identified	0
Literature data	Cyclophosphamide - only older patients	0
	Vincristine - documented DILI	*1*
	Doxorubicin - no documented DILI	0
Exclusion of other Group causes	Group 1 excluded	*1*
	Group 2 excluded (biopsy proven)	0
Reexposure	Not performed	0
Total	—	**3**

DILI, drug-induced liver injury; RUCAM, Roussel Uclaf Causality Assessment Method; HBV, hepatitis B virus.

Other causes of elevated transaminases during chemotherapy include tumor lysis syndrome, viral hepatitis reactivation, and hemophagocytic lymphohistiocytosis, all of which further complicate diagnosis and management. While hepatic infiltration is rare, lymphoblastic involvement should be considered in patients with B-ALL who develop persistent or unexplained transaminase elevations, particularly, when accompanied by hepatomegaly or when other etiologies are excluded.^12,13^ In B-ALL patients with elevated transaminases during chemotherapy, liver biopsy can provide diagnostic clarity and distinguish hepatic infiltration from DILI, avoiding unnecessary interruption of chemotherapy. These cases highlight the importance of a multidisciplinary approach and early consideration of liver biopsy to guide timely management.

## DISCLOSURES

Author contributions: J. Liu, A. Petrosyan, and J. Yoon: Study concept and design, literature search, analysis and interpretation of data, and drafting of the manuscript. M. Roytman: Study supervision and critical revision of the manuscript for important intellectual content. J. Liu is the article guarantor.

Financial disclosure: None to report.

Informed consent was obtained for this case report.

## ABBREVIATIONS:

ALL: acute lymphoblastic leukemia
B-ALL: B-cell acute lymphoblastic leukemia
DILI: drug-induced liver injury
RUCAM: Roussel Uclaf Causality Assessment Method
TdT: terminal deoxynucleotidyl transferase
